# Rhomboid domain-containing protein 1 promotes breast cancer progression by regulating the p-Akt and CDK2 levels

**DOI:** 10.1186/s12964-018-0267-5

**Published:** 2018-10-04

**Authors:** Xin Zhang, Yuechao Zhao, Changjun Wang, Hongge Ju, Wenjie Liu, Xiaohui Zhang, Shiying Miao, Linfang Wang, Qiang Sun, Wei Song

**Affiliations:** 1 Department of Biochemistry and Molecular Biology, State Key Laboratory of Medical Molecular Biology, Institute of Basic Medical Sciences, Chinese Academy of Medical Sciences, Peking Union Medical College, Beijing, 100005 China; 2 Weifang Medical University, Weifang, 261000 China; 3 Department of Breast Surgery, Peking Union Medical College Hospital, Chinese Academy of Medical Science, Beijing, 100730 China; 4 Department of Pathology, Baotou Medical College, Baotou, 014040 China; 5 Department of Pathology, the First Affiliated Hospital of Baotou Medical College, Baotou, 014010 China

**Keywords:** RHBDD1, Breast cancer, P-Akt, CDK2, Cell cycle progression, G1/S phase transition

## Abstract

**Background:**

Our previous work revealed that rhomboid domain-containing protein 1 (RHBDD1) participates in the modulation of cell growth and apoptosis in colorectal cancer cells. This study aimed to investigate the function of RHBDD1 in regulating breast cancer progression and its underlying molecular basis.

**Methods:**

Immunohistochemistry was performed to evaluate RHBDD1 expression in 116 breast cancer tissue and 39 adjacent normal tissue and expression of RHBDD1, phospho-Akt (p-Akt) and cyclin-dependent kinase 2 (CDK2) in the same 84 breast cancer specimens. RHBDD1-knock-out cells were established using breast cancer cell lines. In vitro studies were carried out to estimate the function of RHBDD1 in cell proliferation, migration and invasion. Fluorescence microscopy assay and flow cytometric analysis were used to measure apoptosis and cell cycle regulation. RNA sequencing and western blot analysis were used to investigate the molecular mechanisms of RHBDD1.

**Results:**

RHBDD1 was highly up-regulated in breast cancer tissue compared with that in normal tissue and associated with pathological tumor (pT) stage, pathological tumor-node-metastasis (pTNM) stage and estrogen receptor (ER) expression. RHBDD1 up-regulation was associated with poor prognosis in several subtypes of breast cancer. Deletion of RHBDD1 promoted apoptosis and suppressed proliferation, migration and invasion in breast cancer cells. RHBDD1 deletion suppressed Akt activation and decreased CDK2 protein level via proteasome pathway, thus inhibited cell cycle progression and G1/S phase transition. Moreover, the protein level of RHBDD1, p-Akt and CDK2 was significantly positively correlated in breast cancer tissue.

**Conclusions:**

Our study reveals that RHBDD1 promotes breast cancer progression by regulating p-Akt and CDK2 protein levels, and might be a potential biomarker and prognostic indicator for breast cancer patients.

**Electronic supplementary material:**

The online version of this article (10.1186/s12964-018-0267-5) contains supplementary material, which is available to authorized users.

## Background

Breast cancer is the most commonly diagnosed cancer and the second leading cause of cancer death (19%) in women. In 2016, it is estimated that 30,700 new cases of breast cancer were diagnosed and that 6310 deaths were attributed to breast cancer in the United States [1]. Breast cancer is divided into luminal A, luminal B, HER2-overexpressing and basal-like subtypes according to their differential expression of estrogen receptor (ER), progesterone receptor (PR) and human epidermal growth factor receptor 2 (HER2) [2–6]. Patients with luminal A and luminal B subtypes are hormone receptor positive and sensitive to endocrine therapy [7]. Patients who have the HER2-overexpressing subtype benefit from chemotherapy combined with molecular target therapy [8, 9]. Although these drugs cause a dramatic decrease in mortality, de novo resistance and acquired resistance to these treatments often exist in many breast cancer patients and limit the efficacy of therapeutic strategies [10]. Therefore, numerous studies have focused on identifying new molecular markers of breast cancer and, simultaneously, elucidating the biological mechanisms that cause malignant phenotypes of this disease.

The phosphatidylinositol-4, 5-bisphosphate 3-kinase (PI3K)-Akt signaling pathway plays an important role in breast cancer development. As the most critical signal molecule in this pathway, p-Akt has attracted considerable attention as a therapeutic target [11]. p-Akt is associated with resistance to hormonal treatment in metastatic breast cancer [12], and its expression is significantly higher in ER-positive/HER2-negative ductal carcinoma in situ (DCIS) adjacent to invasive breast cancer (IBC) than pure DCIS [13]. In addition, novel biomarkers and antagonists were reported to regulate p-Akt in breast cancer cells [14, 15].

Rhomboid proteins belong to an intramembrane serine protease family and could be clustered into four major clades: the PARL-type rhomboids, the secretase-type A clade, the secretase-type B clade and the iRhoms group [16, 17]. It has been reported that several human Rhomboids have a strong relationship with carcinogenesis and could serve as prognostic indicators. Human rhomboid family 1 (RHBDF1) was significantly elevated in clinical specimens of invasive ductal breast carcinoma and silencing RHBDF1 with siRNA resulted in apoptosis in breast cancer cells and autophagy in head and neck squamous cancer cells [18]. RHBDD2 was overexpressed in breast cancer cells and was associated with poor prognosis [19]. In our previous work, we determined that RHBDD1 was highly expressed in colorectal cancer tissues but weakly expressed in adjacent normal tissues, and could activate the EGFR/Raf/MEK/ERK signaling pathway through the cleavage and secretion of TGFα as well as up-regulation of EGFR expression [20, 21]. These data suggested that RHBDD1 may be an important tumor biomarker.

In our study, we show that RHBDD1 is up-regulated in breast cancer tissue and closely associated with several important clinicopathological parameters. In addition, we prove that deletion of RHBDD1 results in significant decrease in cell survival, migration, invasion and increase in apoptosis. RHBDD1 deletion suppresses cell cycle progression and G1/S phase transition via down-regulating p-Akt and CDK2 protein levels. Hence, targeting RHBDD1 might be a potential therapeutic strategy for breast cancer treatment.

## Methods

### Tissue microarray (TMA) and immunohistochemical analysis (IHC)

The tissue microarray including tumor tissues and their corresponding adjacent normal tissues from 200 cases of breast cancer was obtained from Shanghai Biochip. The study was approved by the Institutional Review Board of IBMS, CAMS (026–2013). Prior patient consent and approval were obtained for the use of these clinical materials for research purposes. Paraffin-embedded tissue sections (4 μm) were prepared according to classic methods, and the expression of RHBDD1 (1:300 dilution), phospho-Akt (p-Akt; 1:50 dilution) and CDK2 (1:400 dilution) was detected using immunoperoxidase. The expression of RHBDD1, p-Akt and CDK2 was evaluated microscopically and scored according to the intensity and total area of the stain. Slides were assessed by pathologists who did not know the experimental results or patient outcome. The RHBDD1, p-Akt and CDK2 expression was evaluated by immunostaining score, which was calculated as the product of the proportion and intensity of the stain [22]. Briefly, a proportion score was assigned first, which represented the estimated proportion of positive-staining tumor cells (0, none; 1, < 25%; 2, 25–50%; 3, 50–75% and 4, > 75%). Next, an intensity score was assigned, which represented the average intensity of positive tumor cells (0, none; 1, weak; 2, moderate; and 3, strong). The proportion and intensity scores were multiplied together to obtain a single value, ranging from 0 to 12.

### Cell culture and reagents

MDA-MB-231 and MCF7 cells were cultured in Dulbecco’s modified Eagle’s medium (DMEM, HyClone; Thermo Scientific) with 10% fetal bovine serum (FBS), 100 U/mL penicillin and 100 μg/ml streptomycin in 5% CO_2_-humidified incubators at 37 °C. All cell lines were purchased from American Type Culture Collection (ATCC). Lipofectamine 3000 transfection reagent was purchased from Invitrogen. MG132 (S2619) and puromycin (S7417) were purchased from Selleck Chemicals. Thymidine (T1895) was purchased from Sigma-Aldrich. Cycloheximide (CHX; 0970) was purchased from Tocris Bioscience.

### RHBDD1-knockout stable cell lines

The plasmid pX330 (Addgene plasmid 423,230) encodes an optimized hSpCas9 gene and the chimeric guide DNA under mammalian promoters. A pair of oligos for the first exon of RHBDD1 were designed based on the target site sequence, and synthesized by Tsingke (China).The oligos were heated at 95 °C for 5 min, and annealed by decreasing 1 °C/min to 25 °C on a PCR machine (ABI Veriti, USA). The pX330 was digested with Bbs I (Fermentas, Canada) and ligated with the annealed oligos to generate the RHBDD1-knockout plasmid.

MDA-MB-231 and MCF7 cells were transfected with the knockout plasmid using Lipofectamine 3000. After transfection, cells were cultured for 36 h before puromycin (3 μg/ml) was added. Single-cell colonies emerged after two weeks and were examined by PCR and western blot. Positive clones were identified and expanded.

### Antibodies

The RHBDD1 mouse monoclonal antibody was prepared in our laboratory. Its specificity had been verified by Western blot analysis as Song et al. described [20]. Antibodies against Akt (#9272), phospho-Akt (p-Akt; Ser473; #4060P), CDK2 (#2546), CDK4 (#12790), p27 (#3686), CyclinD1 (#2978) and CyclinE1 (#4129P) were purchased from Cell Signaling Technology. GAPDH antibody (sc-47,724) and HRP-conjugated secondary antibodies were purchased from Santa Cruz Biotechnology, Inc.

### Western blot

Proteins were extracted with SDS lysis buffer (50 mM Tris–HCl [pH 6.8], 10% glycerol, 2% SDS) and quantified using BCA protein assay reagent (Thermo Fisher). Extracts were loaded on a 12% SDS–PAGE gel, separated and then electrophoretically transferred to a PVDF membrane (GE Healthcare). The membrane was blocked in 5% skim milk for 1 h at room temperature and then incubated overnight with the indicated antibodies at 4 °C. The membrane was incubated with an anti-rabbit or an anti-mouse HRP-IgG (Santa Cruz) secondary antibody for 1 h at room temperature. Chemiluminescence was detected using an ECL blot detection system (Santa Cruz).

### Cell proliferation assays

Cell proliferation was assessed using the CCK-8 assay (Dojindo Molecular Technologies). 3000 cells per well were seeded in 96-well plates and the medium was replaced every 3 days. Ten microliters of CCK-8 solution was added to each well containing 100 μl of culture medium and incubated for 2 h at 37 °C. The absorbance was measured at 450 nm using an ELISA plate reader. Cell proliferation was measured once per day for seven days.

### Colony formation assays

In total, 1000 cells per well were seeded in 6-well plates. The cells were cultured for 14 days, and visible colonies were formed. The cells were then stained with 0.1% crystal violet, photographed, and the colonies were counted. Each assay was performed in triplicate and repeated three times.

### Migration assay and invasion assay

Migration and invasion assays were performed on Transwell plates. In the migration assay, 1 × 10^5^ MDA-MB-231 cells and 1 × 10^6^ MCF7 cells were seeded on a polycarbonate membrane insert placed in a Transwell plate (Millipore) and cultured in DMEM without serum, and then, DMEM containing 10% FBS was added to the wells of the plates. After incubation at 37 °C in a CO_2_ incubator for several hours (18 h for MDA-MB-231; 36 h for MCF7), the membrane was washed with phosphate-buffered saline (PBS), and the cells at the top of the membrane were wiped with cotton swabs. Cells that migrated to the bottom of the membrane were fixed with methanol, stained with 0.1% crystal violet, and counted in nine random fields at 200× magnification. The invasion assay protocol was similar to that of the migration assay, except that the membrane was coated with 200 ng/mL Matrigel (BD Biosciences).

### Cell cycle and apoptosis assay

For cell cycle analysis, cells were synchronized in G1/S phase by double thymidine (TdR) treatment and prepared for Fluorescence Activated Cell Sorter (FACS) analysis as described [23]. MCF7 cells were blocked using 2 mM TdR for 18 h, followed by 12 h (for wild-type cells) or 13 h (for RHBDD1-knockout cells) release in fresh medium and then 18 h further incubation with 2 mM TdR. MDA-MB-231 cells were blocked using 2 mM TdR for 17 h, followed by 9 h (for wild-type cells) or 12 h (for RHBDD1-knockout cells) release in fresh medium and then 17 h further incubation with 2 mM TdR. Cells were released in fresh medium after two washes in PBS. Cells were stained with propidium iodide (PI; MCH100106; Millipore) according to the manufacturer’s instructions and DNA content was examined by FACS analysis with default parameters. The results were analyzed using ModFit software.

To detect apoptosis, an Annexin V/7AAD double staining kit (MCH100105; Millipore) was used to stain cells according to the manufacturer’s instructions. Apoptotic cells were determined by FACS analysis.

### TdT-mediated dUTP nick-end labeling (TUNEL) assay

An In Situ Cell Death Detection Kit purchased from Roche was used to detect apoptosis according to the manufacturer’s instructions. Briefly, approximate 6000 cells per well were seeded in 96-well plates. Three days after being plated, the cells were washed with PBS twice and fixed in freshly prepared 4% paraformaldehyde in PBS (pH 7.4) for 1 h. Then, the cells were permeabilized in freshly prepared 0.1% Triton X-100 (in 0.1% sodium citrate) for 5 min on ice. Finally, cells were added to 50 μl of TUNEL reaction mixture per well, incubated for 1 h at 37 °C, and analyzed by fluorescence microscopy.

### Quantitative real-time PCR

Total RNA was isolated from different cell lines using TRIZOL Reagent (Invitrogen) according to the manufacturer’s instructions. Equal amounts of RNA were reverse transcribed into cDNA using PrimeScript RT Master Mix (TaKaRa) as instructed by the manufacturer. Quantitative real-time PCR reaction was performed using SYBR Green Power Master Mix (Applied Biosystems). The primers that were used are shown in Additional file 1: Table S1.

### cDNA library preparation and sequencing

The sequencing library of each RNA sample was prepared using the Illumina TruSeq RNA Library Prep Kit v2 according to the protocol provided by the manufacturer (Illumina, USA) [24]. FAST-QC was used to evaluate the quality of the sequencing data [25]. After quality testing, raw reads were mapped to the reference human genome using the HISAT2 algorithm with default parameters [26]. A supplementary table showed the data of differentially expressed genes in more detail (Additional file 2: Table S2).

### Hierarchical clustering and gene set enrichment analysis

Hierarchical clustering was performed as described using Cluster version 3.0 and Java TreeView version 1.1.6 to identify and visualize the DEGs derived from analysis of apoptosis (GO ID = 0006915) and cell cycle (GO ID = 0007049), respectively, in the Gene Ontology database [27–29]. Gene Set Enrichment Analysis (GSEA) was performed using the GSEA Java desktop software application (Broad Institute) [30]. Results with *p* < 0.05 and FDR < 0.05 were considered statistically significant [31].

### Statistical analysis

Clinical data were analyzed using the R software [32]. Wilcoxon rank-sum test was applied to analyze RHBDD1 expression between breast cancer tissues and normal tissues, as well as the difference of p-Akt and CDK2 expression between weak and strong RHBDD1 expression group [33]. Clinicopathological parameters were evaluated using the Fisher’s exact test and Pearson’s chi-squared test. Relapse-free and overall survival were analyzed using the resources published in the Kaplan Meier plotter (http://kmplot.com/, Affi id = 226945_at) by the Kaplan–Meier method with log-rank testing [34]. Spearman’s rank correlation test was used to analyze the clinical correlation of RHBDD1, p-Akt and CDK2 [35]. Statistical analysis of experimental data was performed using GraphPad prism 5.0. The results were statistically evaluated using a t test and the ANOVA test. *p* < 0.05 was considered statistically significant.

## Results

### RHBDD1 is highly up-regulated in breast cancer tissue

**Fig. 1:**
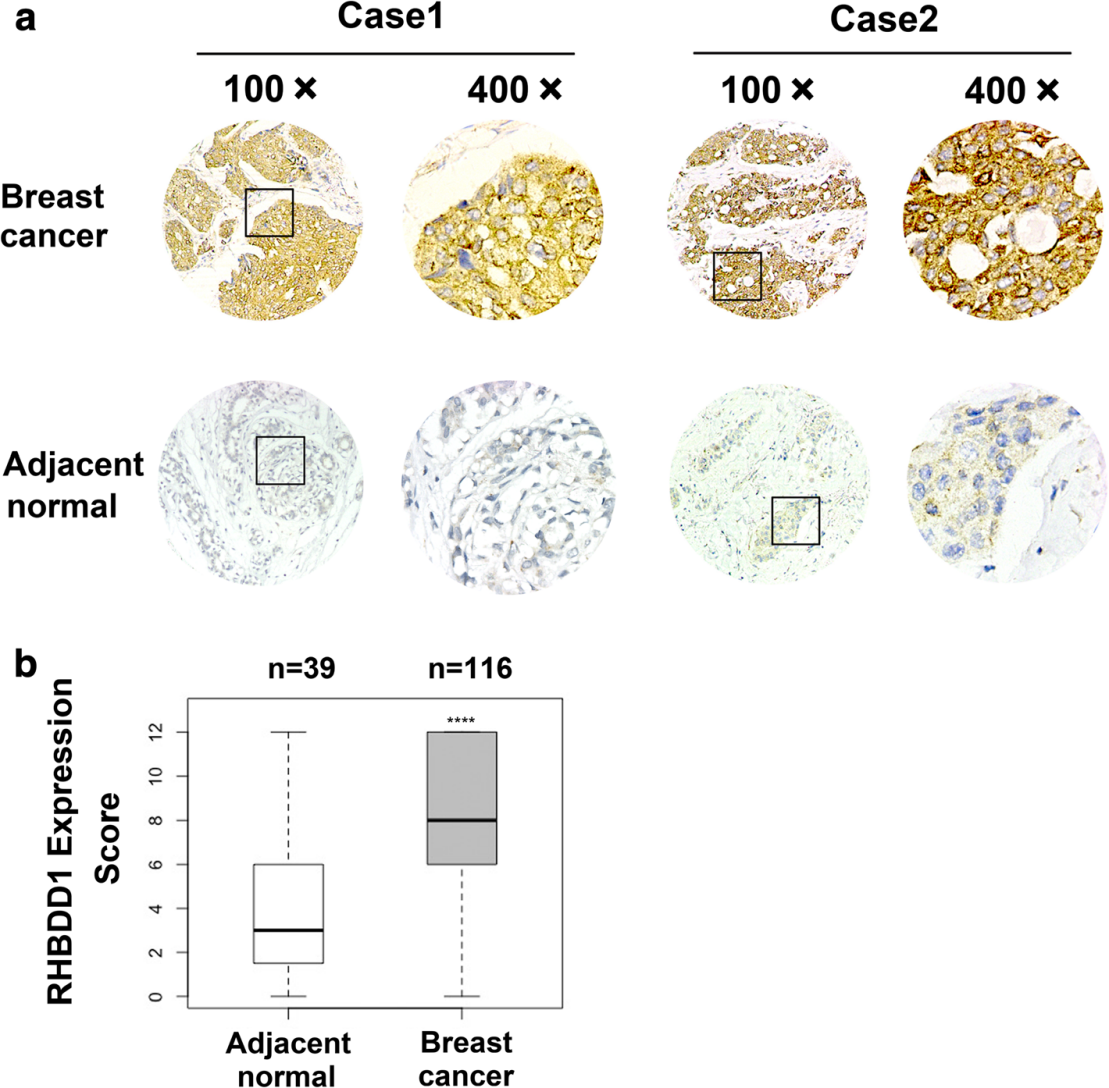
RHBDD1 expression in specimens from breast cancer patients. **a** Representative photos of immunohistochemical staining of a breast cancer tissue microarray. Breast cancer samples with high scores and normal tissue samples with low scores are shown. Original magnification, 100×; 400×. **b** Tissue microarray data analysis of RHBDD1 expression in 116 breast cancer samples (gray) and 39 adjacent normal samples (white) (Wilcoxon rank-sum test, **** *p* < 0.0001)

We performed immunohistochemical staining to assess RHBDD1 protein expression levels in 116 breast cancer specimens and 39 matched adjacent normal breast tissue samples (Fig. 1a and b, Table 1). As Fig. 1a. showed, the immunohistochemical data revealed that in breast tumor tissue, the cellular localization of RHBDD1 was restricted to both plasma membrane and cytoplasm. This was in accordance with our previous results in colorectal tumors and validated the cellular localization of RHBDD1 in tumor cells [20, 21]. RHBDD1 was highly up-regulated in breast cancer tissue than adjacent normal tissue (*p* = 1.566e-12). The median expression was 2.7-fold higher in tumor than in normal breast tissue (Fig. 1a and b). When considering a sample having an IHC score of 5 or higher to have strong RHBDD1 expression, 94/116 (81%) of breast cancer specimens were classified as having strong RHBDD1 expression, while only 11/39 (28%) of normal tissue had strong RHBDD1 expression.

**Table 1:** Association between RHBDD1 expression and clinicopathologic parameters in 116 breast cancer patients

Clinicopathologic parameters	N	RHBDD1		*p*-value
		Weak (IHC Score < 5)	Strong (IHC Score ≥ 5)	
Age				0.6357
≤ 50	58	12	46	
> 50	58	10	48	
Differentiation				0.2066
Low	7	3	4	
Moderate	106	19	87	
High	3	0	3	
pTNM				0.01474*
I	6	3	3	
II	67	8	59	
III	39	11	28	
pT				1.165e-13*
T1	24	6	18	
T2	79	13	66	
T3	12	2	10	
pN				0.1734
N0	41	7	34	
N1	35	4	31	
N2	28	8	20	
N3	8	3	5	
ER				0.04679*
Negative	42	12	30	
Positive	74	10	64	
PR				0.2443
Negative	50	12	38	
Positive	65	10	55	
HER2				0.7027
Negative	88	16	72	
Positive	28	6	22	

*pTNM* pathological tumor-node-metastasis, *pT* pathological tumor, *pN* pathological node

The significance of RHBDD1 expression in clinicopathologic parameters was analyzed by Pearson’s chi-squared test. If the expected counts were less than 5, Fisher’s exact test was used to analyze the statistics (* *p* < 0.05)

We further evaluated the possible correlation between RHBDD1 expression and clinicopathologic parameters (Table 1). Statistical analysis showed that elevated RHBDD1 levels were remarkably associated with pT stage (*N* = 115, *p* = 1.165e-13), pTNM stage (*N* = 112, *p* = 0.01991) and ER expression (*N* = 116, *p* = 0.04679). However, RHBDD1 expression was not associated with other parameters, such as age, differentiation, pathological node stage (pN), PR expression, HER2 expression.

Besides, we analyzed the correlations between RHBDD1 expression and relapse-free survival (RFS) and/or overall survival (OS) to determine whether RHBDD1 expression level in tumors is associated with prognosis. We found that patients with low RHBDD1 expression had better RFS or OS times in ER positive breast cancer, ER and PR positive breast cancer, HER2 positive breast cancer, PR positive breast cancer and triple negative breast cancer (the Kaplan–Meier method with log-rank testing, Additional file 3: Figure S1). These data suggest that RHBDD1 may be a potential prognostic indicator in several subtypes of breast cancer.

### Deletion of RHBDD1 suppresses breast cancer cell survival, migration and invasion

**Fig. 2:**
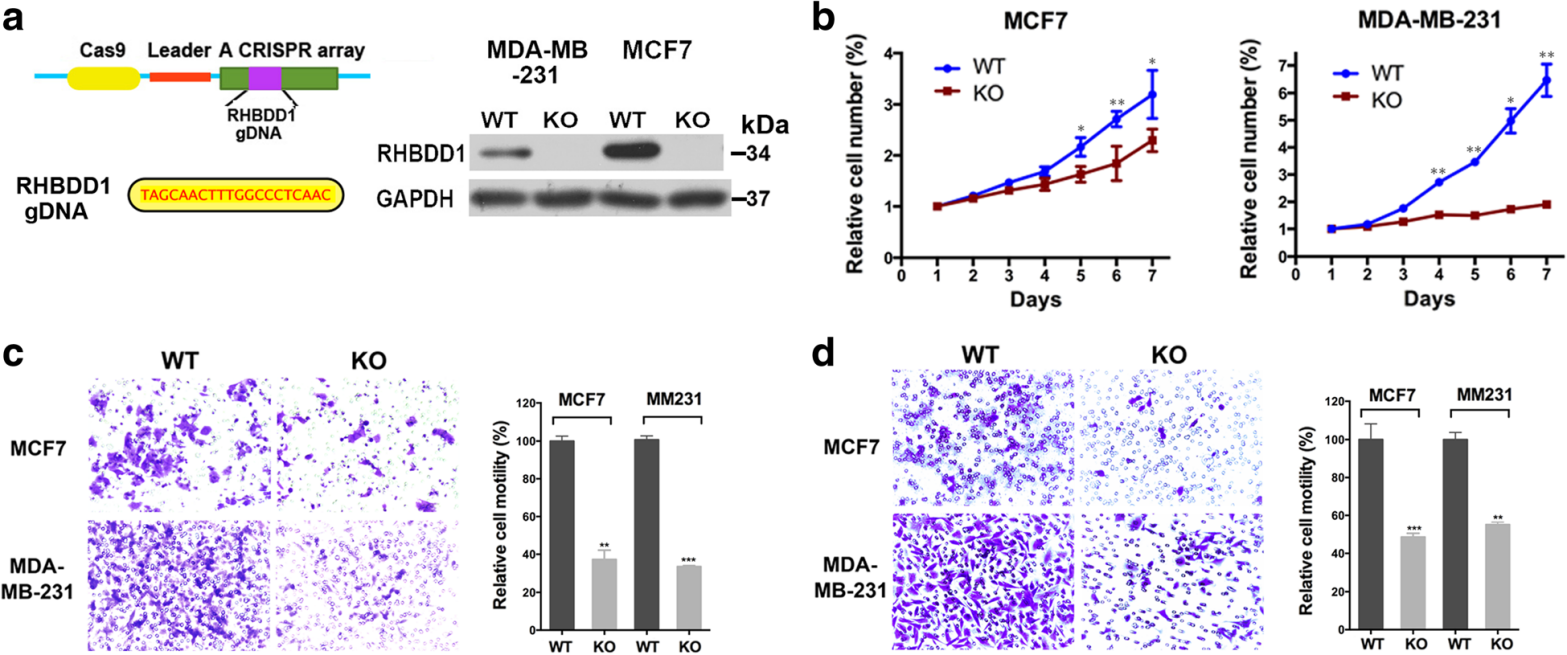
The effect of RHBDD1 deletion on proliferation, migration and invasion in breast cancer cells. **a** CRISPR/Cas9-mediated RHBDD1-knockout system. MCF7 and MDA-MB-231 RHBDD1-knockout cells exhibited no RHBDD1 expression as determined by western blotting. GAPDH was a loading control. Experiments were repeated four times. **b** Cell proliferation assays. Each point represented the mean value of five independent samples. Experiments were repeated three times. **c**. and **d**. Representative photos and statistical plots of migration assays and Matrigel chemoinvasion assays. Original magnification, 200× (means±s.d., t test, ** *p* < 0.01; *** *p* < 0.001). Experiments were repeated three times

Using the CRISPR/Cas9 genome editing system, we knocked out RHBDD1 in triple-negative MDA-MB-231 cells and estrogen receptor-positive MCF7 cells (Fig. 2a) [36]. As shown in Fig. 2b, deletion of RHBDD1 significantly reduced the growth rate in both MDA-MB-231 and MCF7 cells. In contrast, reduced expression of RHBDD1 by knock-down experiment did not affect the proliferation rate of non-tumor HEK 293 T cells (Additional file 4: Figure S2). Colony number and average colony size were remarkably lower in RHBDD1-knock-out cells than in wild-type MDA-MB-231 and MCF7 cells (Additional file 5: Figure S3). Besides, transwell migration assays and invasion assays revealed that RHBDD1 deletion inhibited cell movement to the bottom of the chamber in MDA-MB-231 and MCF7 cells (Fig. 2c and d).

### Apoptosis in breast cancer cells increases in the absence of RHBDD1

**Fig. 3:**
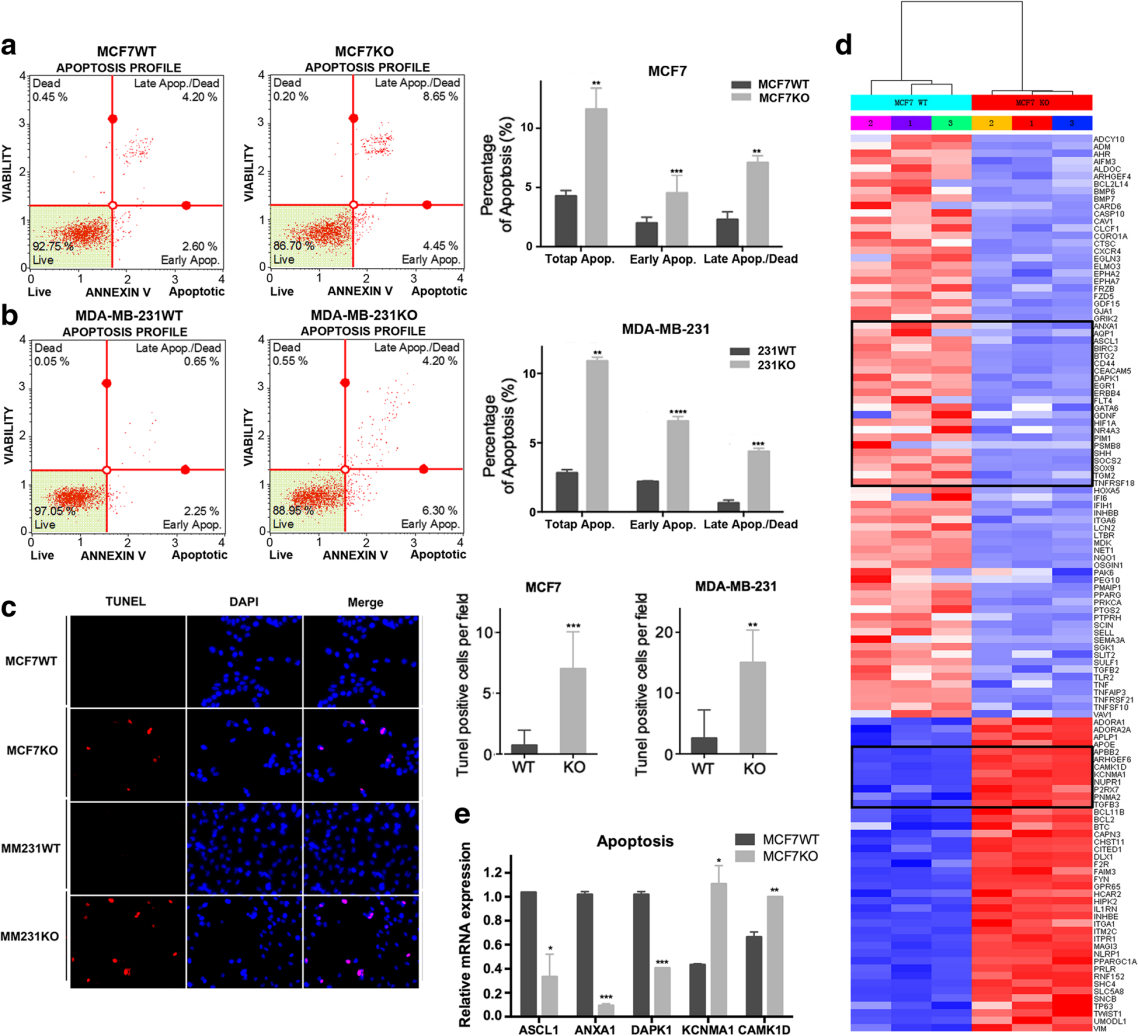
RHBDD1 deletion induces apoptosis in breast cancer cells. **a** Representative flow cytometry plots and statistical plots from triplicate apoptosis assays of MCF7 wild-type and knock-out cells (means±s.d., t test, ** *p* < 0.01; ***p < 0.001). Experiments were repeated three times. **b** Representative flow cytometry plots and statistical plots from triplicate apoptosis assays of MDA-MB-231 wild-type and knock-out cells (means±s.d., t test, ** *p* < 0.01; ***p < 0.001). Experiments were repeated three times. **c** Representative photos of the TUNEL assay and statistical plots from triplicate assays. Original magnification, 50× (means±s.d., t test, ** *p* < 0.01; ***p < 0.001). Experiments were repeated three times. **d** Heatmap of apoptosis related genes differentially expressed in MCF7 wild-type and knock-out cells using R software. The log2 values were calculated for each sample by normalizing to the number of reads alone (*p* < 0.05, FDR < 0.05). **e**. Relative mRNA expression of ASCL1, ANXA1, DAPK1, KCNMA1 and CAMK1D in MCF7 wild-type and knock-out cells determined by quantitative real-time PCR and normalized to GAPDH (means±s.d., t test, * *p* < 0.05). Experiments were repeated three times

To determine whether RHBDD1 deletion increases apoptosis, we conducted three sets of experiment. First, we tested the percentage of apoptosis in RHBDD1-knock-out and wild-type cells using FACS analysis. For MCF7 cells, the percentage of total apoptotic cells increased from 4.27% (wild-type) to 11.6% (knock out), and the percentages of early apoptosis and late apoptosis increased from 1.98% (wild type) and 2.28% (wild type) to 4.52% (knock out) and 7.08% (knock out), respectively (Fig. 3a). The tendencies of MDA-MB-231 cells were similar to those of MCF7 cells. The proportion of total apoptosis increased from 2.82% (wild-type) to 10.9% (knock out), and the proportion of early apoptosis and late apoptosis increased from 2.18% (wild type) and 0.63% (wild type) to 6.53% (knock out) and 4.37% (knock out), respectively (Fig. 3b). Second, apoptosis was further evaluated by fluorescence microscopy assay. The number of apoptotic cells increased significantly in MCF7 and MDA-MB-231 knock-out cells, at 9.8-fold and 5.8-fold higher than MCF7 and MDA-MB-231 wild-type cells, respectively (Fig. 3c). In the third experiment, RNA sequencing was performed using 3 MCF7 wild-type cell lines and 3 RHBDD1-knockout cell lines to investigate the transcription levels of apoptosis related genes. We analyzed differentially expressed genes and constructed a heatmap. As shown in Fig. 3d, compared with wild-type cells, 120 apoptosis related genes were differentially expressed in MCF7 RHBDD1-knockout cells (*p* < 0.05), including 42 upregulated genes and 78 downregulated genes. According to the KEGG annotation, we observed that 8 upregulated genes promoted the apoptotic process and 22 downregulated genes inhibited the apoptotic process (Additional file 6: Table S3) [37]. mRNA levels of several randomly picked-up genes were tested by qRT-PCR to confirm the heatmap data (Fig. 3e). The mRNA levels of 3 downregulated genes (ASCL1, ANXA1 and DAPK1) were more than 2-fold lower in RHBDD1-knockout cells than in wild-type cells. Conversely, mRNA levels of 2 upregulated genes (KCNMA1 and CAMK1D) were remarkably elevated (~ 2-fold) in RHBDD1-knockout cells. These results were consistent with the heatmap data. Taking these results together, we determined that RHBDD1 deletion increased apoptosis in breast cancer cells.

### Deletion of RHBDD1 suppresses proper cell cycle progression and G1/S phase transition

**Fig. 4:**
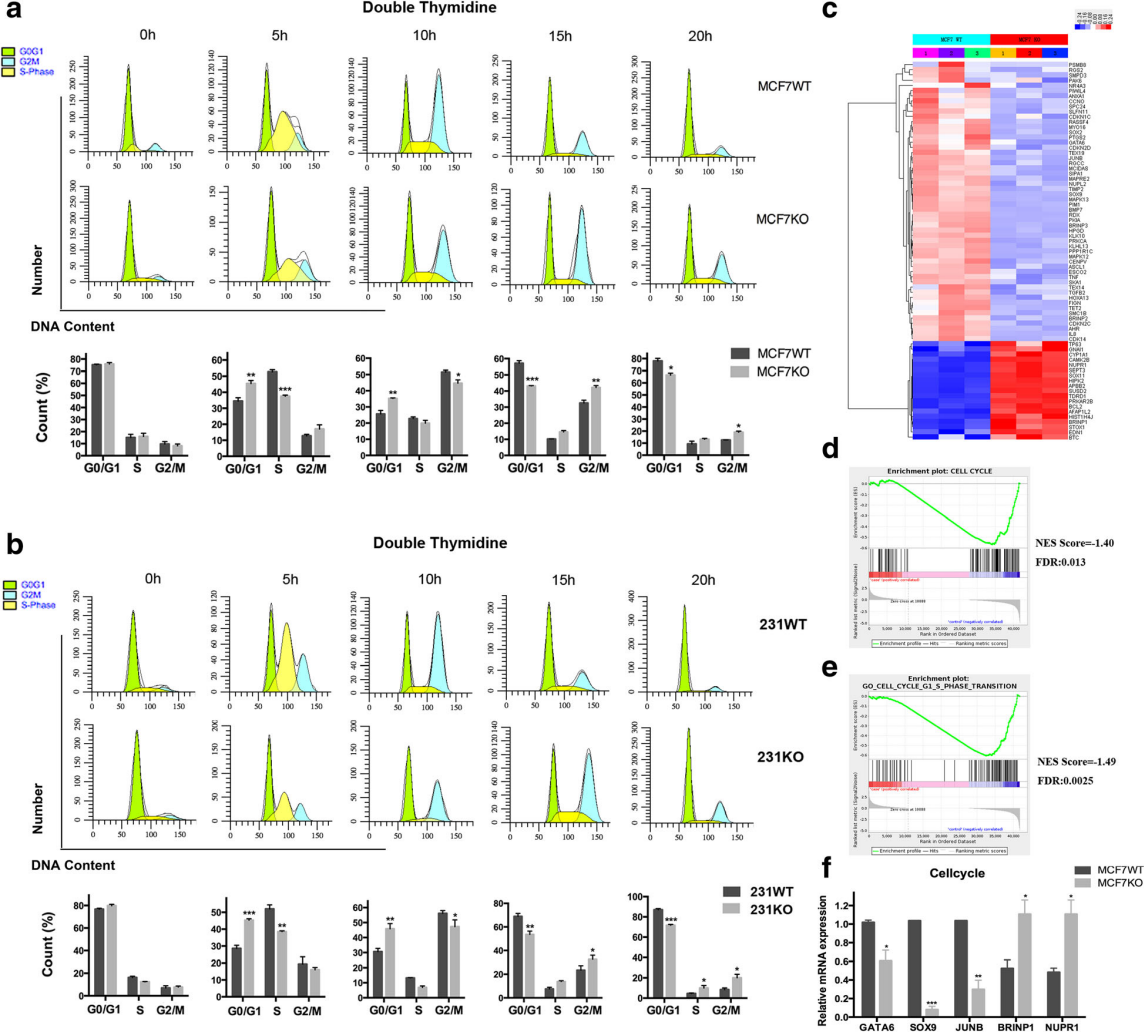
RHBDD1 deletion suppresses cell cycle progression and blocks G1/S transition. **a** DNA content of wild-type and knock-out MCF7 cells were determined at indicated time points after double thymidine treatment (means±s.d., two-way ANOVA, * *p* < 0.05). Experiments were repeated five times. **b** DNA content of wild-type and knock-out MDA-MB-231 cells were determined at indicated time points after double thymidine treatment (means±s.d., two-way ANOVA, * *p* < 0.05). Experiments were repeated five times. **c** Heatmap of cell cycle related genes differentially expressed in MCF7 wild-type and knock-out cells using R software. The log2 values were calculated for each sample by normalizing to the number of reads alone (*p* < 0.05, FDR < 0.05). **d** Gene Set Enrichment Analysis (GSEA) of genes involved in cell cycle progression in MCF7 RHBDD1 knock-out cells with the gene sets corresponding to CELL CYCLE in the KEGG database (NES Score = −1.40, FDR = 0.013). **e** GSEA of genes involved in G1/S phase transition in MCF7 RHBDD1-knockout cells with the gene sets corresponding to GO_CELL_CYCLE_G1_S_PHASE_TRANSITION in the MSigDB database (NES Score = − 1.49, FDR = 0.0025). **f** Relative mRNA expression of GATA6, SOX9, JUNB, BRINP1 and NUPR1 in MCF7 wild-type and knock-out cells by quantitative real-time PCR, normalized to GAPDH (means±s.d., t test, * *p* < 0.05). Experiments were repeated three times

Next, we tested whether RHBDD1deletion has an effect on cell cycle progression. A double thymidine synchronization system was used to arrest RHBDD1-knockout and wild-type MCF7 and MDA-MB-231 cells at the border between the G1 and S phase. We examined the cell cycle phases at different time points using FACS analysis. At 0 h, most cells accumulated at the border between the G1 and S phase in both knock-out and wild-type MCF7 cells (75% and 76%, respectively, Fig. 4a). In control cells, at 5 h after synchronization, the percentage of cells in the G1 phase was remarkably reduced (34.5%), and a large proportion of cells progressed into the S phase (52%). In contrast, RHBDD1-knockout cells showed a slower decrease in the proportion of cells in the G1 phase (46.2%) (*p* < 0.0001), and thus, a significantly reduced percentage of cells were in the S phase (38.6%) (*p* < 0.001). At 10 h, 25.7% of control cells and 36.3% of knock-out cells were still in the G1 phase (*p* < 0.001). The proportion of cells in G2/M phase began to accumulate, with 51.5% of control cells and 44.7% of knock-out cells in this phase (*p* < 0.01) (Fig. 4a). At 15 h, a large number of control cells re-entered into the G1 phase (57.3%), while knock-out cells showed a significantly reduced G1 population (44.2%) (*p* < 0.0001). The percentages of cells in the G2/M phase were 31.7% and 42.9%, in control and knock-out cells, respectively (*p* < 0.001, Fig. 4a). At 20 h, 79.1% of control cells re-entered into the G1 phase, which was higher than the 66.9% of knock-out cells in this phase (*p* < 0.01, Fig. 4a).

The findings in MDA-MB-231 cells were consistent with the results in MCF7 cells. As shown in Fig. 4b, at 5 h, 45.4% of knock-out cells were still blocked in the G1 phase and 37.5% entered into the S phase. While for wild-type cells, 28.6% remained in the G1 phase, and 52.8% progressed into the S phase. At 10 h after synchronization, we observed that 30.6% of the control cells remained in the G1 phase, which was lower than the 45.8% of knock-out cells in this phase (*p* < 0.01). At 20 h, 87% of the control cells re-entered into the G1 phase, which was remarkably higher than the 71.7% of knock-out cells in this phase (*p* < 0.0001, Fig. 4b).

Furthermore, we performed RNA-seq analysis of all the cell cycle related genes (according to KEGG annotation).The heatmap revealed that 73 genes were differentially expressed between RHBDD1 knock-out and wild-type MCF7 cells (*p* < 0.05), and they were classified into two main clusters, including 19 upregulated genes and 54 downregulated genes (Fig. 4c, Additional file 7: Table S4). QRT-PCR analysis was performed using RHBDD1 knock-out and wild-type MCF7 cells to test the transcript levels of 5 randomly picked-up genes. As shown in Fig. 4f, the transcript levels of GATA6, SOX9 and JUNB, 3 down-regulated genes revealed on the heatmap, increased nearly 2-fold in knock-out cells, whereas the transcript levels of BRINP1 and NUPR1, 2 upregulated genes revealed on the heatmap, decreased more than 2-fold in knock-out cells (Fig. 4f). These results were in accordance with the heatmap data.

Gene Set Enrichment Analysis (GSEA) of all the cell cycle related genes demonstrated significant enrichment for genes downregulated in RHBDD1 knock-out MCF7 cells as compared with the gene sets corresponding to CELL CYCLE (pathway:map04110) in the KEGG database (NES Score = − 1.40, FDR = 0.013, Fig. 4d). GSEA of all the genes involved in G1/S phase transition demonstrated significant enrichment for genes downregulated in RHBDD1 knock-out MCF7 cells as compared with the gene sets corresponding to GO_CELL_CYCLE_G1_S_PHASE_TRANSITION (GO ID = 0044843) in the MSigDB database (NES Score = − 1.49, FDR = 0.0025, Fig. 4e) [38]. Both results indicated that the cell cycle progression and G1/S phase transition may be suppressed in MCF7 RHBDD1-knock-out cells.

The molecular mechanism of RHBDD1 to modulate the cell cycle and G1/S transition

**Fig. 5:**
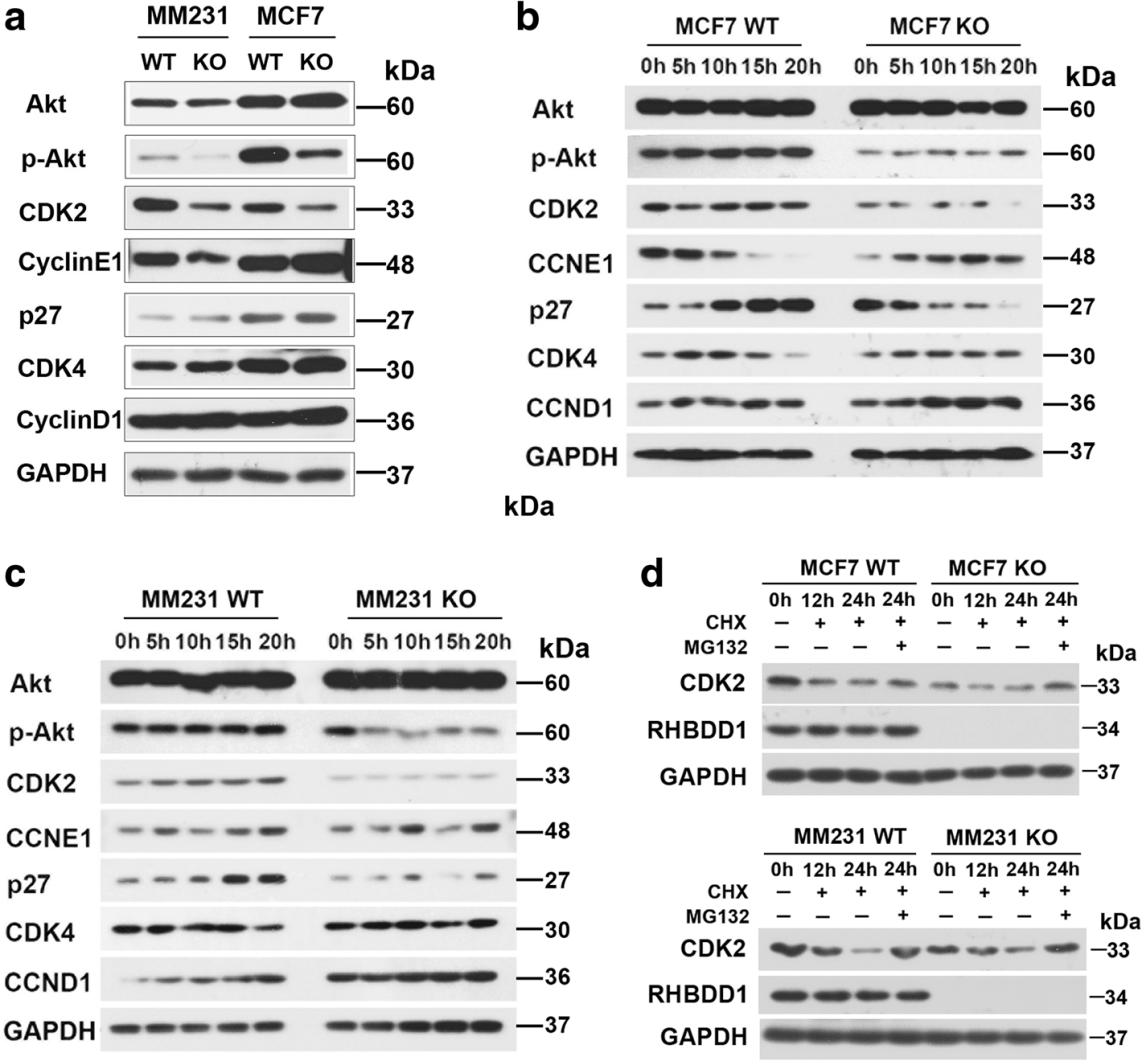
RHBDD1 deletion decreases the protein levels of p-Akt and CDK2. **a** The protein levels of Akt, p-Akt, CDK2, cyclinE1, p27, CDK4 and cyclinD1 were analyzed by western blot using GAPDH as a loading control. Experiments were repeated three times. **b** Western blot analysis for Akt, p-Akt, CDK2, cyclinE1, p27, CDK4 and cyclinD1 expression in MCF7 wild-type and knock-out cells at the indicated time points after double thymidine treatment. GAPDH was a loading control. Experiments were repeated three times. **c** Western blot analysis for Akt, p-Akt, CDK2, cyclinE1, p27, CDK4 and cyclinD1 expression in MDA-MB-231 wild-type and knock-out cells at indicated time points after double thymidine treatment. GAPDH was a loading control. Experiments were repeated three times. **d** CDK2 protein levels of wild-type and knock-out MCF7 and MDA-MB-231 cells were analyzed by western blot assay. Cells were treated with 10 μM CHX, along with MG132 (1 μM for MCF7 and 2 μM for MDA-MB-231) or vehicle DMSO control, and samples were collected at the indicated times. Both RHBDD1 and GAPDH were loading controls. Experiments were repeated three times

Having observed improper cell cycle progression and the G1/S phase arrest, we further investigated whether RHBDD1 deletion altered the signaling pathway associated with cell cycle and G1/S phase transition. We examined the effect of RHBDD1 on 7 G1/S transition related proteins: CDK2 [39], cyclinE1 [40], p27 [41], CDK4 [42], cyclinD1 [43], Akt and p-Akt. p-Akt is an important signal molecule in the PI3K-Akt pathway involved in G1/S regulation [44]. Western blot analysis showed that p-Akt and CDK2 protein levels were remarkably decreased in RHBDD1-knockout cells in the MDA-MB-231 and MCF7 cell lines. No difference was observed in the protein levels of other cell cycle regulators, including Akt, cyclinE1, p27, CDK4 and cyclinD1 (Fig. 5a). After double thymidine treatment, the protein levels of p-Akt and CDK2 were notably reduced at different time points in RHBDD1-knockout cells compared to those in the wild-type cells, whereas no remarkable changes were detected in other cell cycle regulators (Fig. 5b and c).

RNA- sequencing analysis detected no marked change in the transcripts of these proteins between wild-type and knock-out cells (Fig. 4c). In mammalian cells, cellular proteins are often degraded in the ubiquitin-proteasome pathway [45]. We speculated that CDK2 might be partially degraded by a proteasome. We treated the cells with CHX to stop translation, along with MG132, an effective inhibitor of proteasomes, or DMSO as control [46]. CDK2 protein levels at the indicated time points (0 h, 12 h and 24 h after treatment) were monitored by western blot assay. 24 h’ treatment of MG132 remarkably restored CDK2 protein levels in both MDA-MB-231 and MCF7 cell lines (Fig. 5d).

### RHBDD1 expression has a positive correlation with p-Akt and CDK2 in breast cancer tissues

**Fig. 6:**
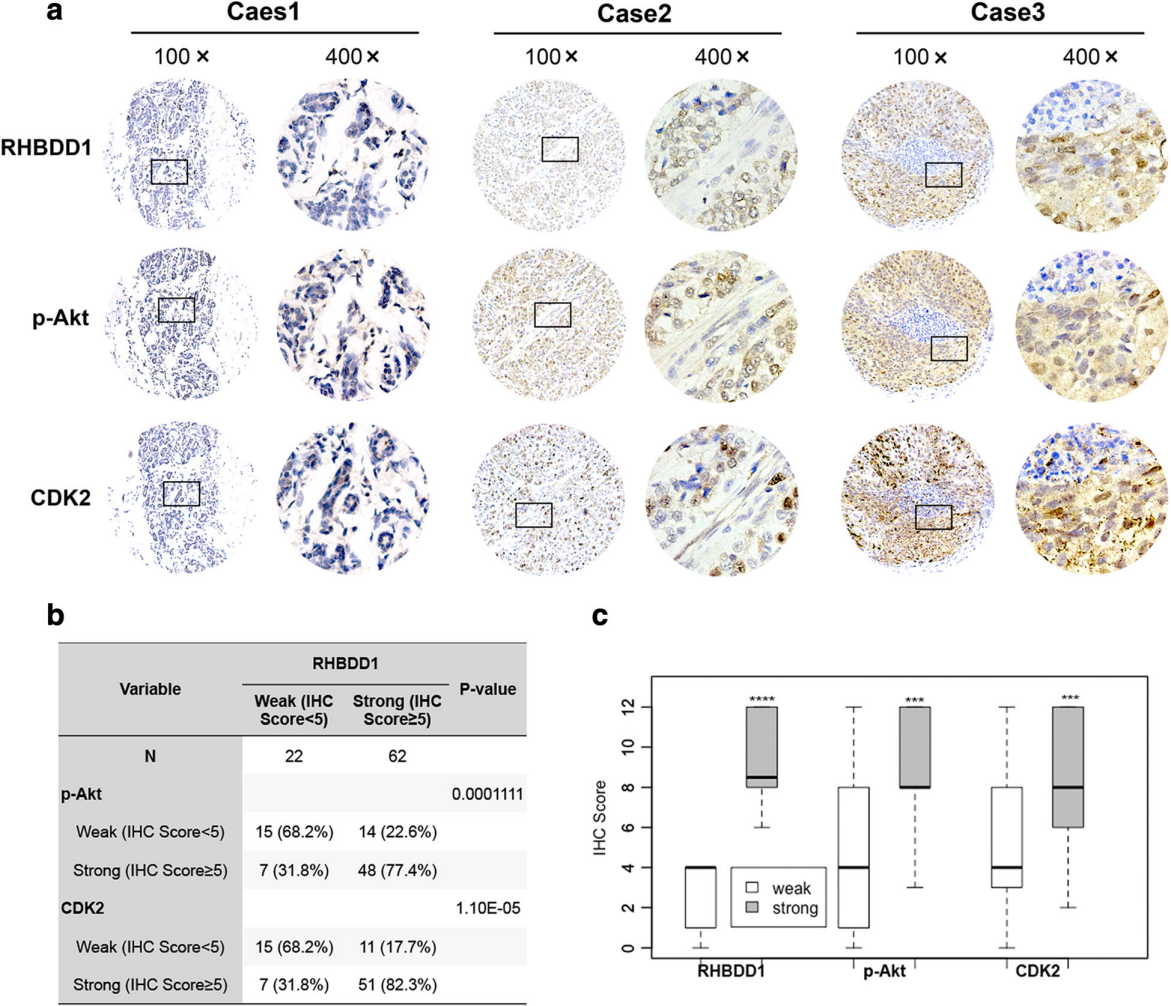
RHBDD1, p-Akt and CDK2 expression are positively correlated in breast cancer tissues. **a** The expression of RHBDD1, p-Akt and CDK2 in 84 breast cancer tissue samples were detected by immunohistochemistry. Representative photos showed the consistent expression of RHBDD1, p-Akt and CDK2. Case 1: weak staining; Case 2: moderate staining; Case 3: strong staining. Original magnification, 100×; 400×. **b** The protein levels of p-Akt and CDK2 were associated with RHBDD1 levels (Pearson’s chi-squared test, *p* < 0.001). **c** Boxplots show the consistent expression of RHBDD1, p-Akt and CDK2 in the RHBDD1 weak expression group (white) and the RHBDD1 high expression group (gray) (Wilcoxon rank-sum test, *** *p* < 0.001)

To confirm the relationship between RHBDD1, p-Akt and CDK2 observed in the above results, we detected and analyzed the levels of the three proteins in 84 breast cancer specimens by IHC using serial sections from the same TMAs (Fig. 6a, Additional file 8: Table S5). Pairwise comparison using Spearman’s rank correlation test revealed that the protein level of RHBDD1 and p-Akt was remarkably positively correlated in breast cancer tissue (Spearman’s ρ = 0.437; *p* = 3.245e-05), as was that of RHBDD1 and CDK2 (Spearman’s ρ = 0.386; *p* = 0.0003). In addition, there was a significant positive correlation between CDK2 and p-Akt (Spearman’s ρ = 0.421; *p* = 6.613e-05). Representative photos of breast cancer tissue with different expression intensities for RHBDD1, p-Akt and CDK2 are shown in Fig. 6a. According to the IHC scores of RHBDD1 expression, we divided all 84 specimens into two groups: 22 tumors were in the RHBDD1 weak expression group, with IHC scores of 0–4, and 62 tumors were in the RHBDD1 strong expression group, with IHC scores of ≥5 (Fig. 6b and c). As shown in Fig. 6b, RHBDD1 was closely associated with p-Akt and CDK2 (*N* = 84, *p* < 0.001). Weak levels (IHC scores< 5) of p-Akt and CDK2 were observed in 68.2% (15/22) of tumors in the RHBDD1 weak expression group, whereas in the RHBDD1 strong expression group, 77.4% (48/62) and 82.3% (51/62) of tumors exhibited high levels (IHC scores≥5) of p-Akt and CDK2, respectively [47]. In the RHBDD1 weak expression group, a median IHC score of 4 was reported for RHBDD1, p-Akt and CDK2. In the RHBDD1 strong expression group, the median IHC score for RHBDD1 was 8.5, whereas p-Akt and CDK2 had median IHC scores of 8 (Fig. 6c). These values were approximately equivalent to each other. As shown in Fig. 6c, the difference in protein levels between the two groups was significant for RHBDD1, p-Akt and CDK2 (*p* < 0.001).

## Discussion

RHBDD1 was first identified to be involved in mammalian spermatogenesis and belonged to the secretase-type B clade according to the phylogenetic analysis [17, 48]. It had been reported that RHBDD1 has pleiotropic cellular effects. For example, RHBDD1 is an important protease and cleaves several different substrates, including BIK, a proapoptotic member of the Bcl-2 family, and the amyloid precursor protein, a key molecule in the etiology of Alzheimer disease [48, 49]. Another famous biological role of RHBDD1 is that it may participate in the endoplasmic reticulum (ER) quality control system. Fleig et al. reported that, in Hek293T cells, RHBDD1 promotes ER-associated degradation (ERAD) through the cleavage of its ERAD substrates, which are various ubiquitinated single-spanning and polytopic membrane proteins with positively charged transmembrane residues [50].

Our previous work showed that RHBDD1 expression is elevated in colorectal cancer tissue and associated with poor prognosis [20]. In the current study, we proved that RHBDD1 is highly up-regulated in breast cancer tissue than adjacent normal tissue (Fig. 1b). This tumor-highly expression pattern offers an important clue that RHBDD1 is important in tumorigenesis and might be used as a novel biomarker to identify breast cancer patients. Besides, RHBDD1 upregulation is associated with poor prognosis in ER positive breast cancer, ER and PR positive breast cancer, HER2 positive breast cancer, PR positive breast cancer and triple negative breast cancer.

RHBDD1 expression level is remarkably associated with the pT stage, pTNM stage and ER expression. These clinicopathological results are consistent with our in vitro data, which showed that RHBDD1 deletion remarkably inhibits breast cancer cell proliferation and migration/invasion, respectively, in both ER-positive MCF7 and Triple-negative MDA-MB-231 cell lines. The growth curves indicated that the effect of RHBDD1 deletion differed modestly between the two cell lines, and a significant decrease in growth rate was observed in MDA-MB-231 knock-out cells compared with wild-type cells, although RHBDD1 levels exhibited no significant association with triple-negative breast cancer (TNBC) in clinical specimens.

We further observed that reduced RHBDD1 expression has no effect on the proliferation rate of non-tumor cell line as compared with tumor cell lines. We speculate that one mechanism may involve the different expression level of RHBDD1 in tumor and normal cell lines. Besides, we proposed that the inhibitory effect on breast cancer cell growth caused by RHBDD1 deletion may be attributed to increased cell apoptosis or improper cell cycle progression or both processes. Previous work showed that RHBDD1 could inhibit cell apoptosis by activating and upregulating c-Jun and its downstream target, Bcl-3, and this inhibitory effect occurred under different apoptotic stimuli but not under normal condition [51]. Consistently, in our present study, we also detected that loss of RHBDD1 could markedly increase apoptosis of breast cancer cells. Moreover, RNA-seq data of wild-type and knock-out MCF7 cells suggests that RHBDD1 deletion regulates large amounts of apoptosis-related genes which are annotated in the KEGG database (Fig. 3d). Although the accurate mechanism is still unknown, it merits further investigation. Additional western blot experiment and IHC analysis needs to be carried out to confirm the RNA-seq result. Moreover, we observed that RHBDD1 deletion blocks G1/S phase transition and delays breast cancer cell cycle progression after synchronization with TdR. Our work showed that exit from G1 into S phase was delayed and the G1 population at 5 h after TdR treatment significantly increased in RHBDD1-knockout cells, compared with wild-type cells (*p* < 0.001). The average cell cycling rate in MCF7 and MDA-MB-231 knock-out cells decreased ~ 11% and ~ 16%, respectively, compared with wild-type cells (*p* < 0.01). GSEA results confirmed enrichment of downregulated-genes in G1/S phase transition and cell cycle progression in MCF7 knock-out cells. Taken together, we proposed that the growth inhibition of breast cancer cells caused by RHBDD1 deletion may largely be attributed to RHBDD1-mediated modulation of cell apoptosis and cell cycle.

As critical regulators, CDKs, cyclins and their inhibitors, such as p27, should be coordinated to perfectly maintain proper cell cycle and G1/S progression [52]. CDK2 forms a complex with cyclinE1 to promote G1/S transition and forms a complex with cyclinA to bypass the S phase and the G2/M phase [53]. RNA sequencing analysis revealed that mRNA levels of CDK2 are not significantly altered in either RHBDD1-knockout or wild-type cells. We observed that RHBDD1 deletion significantly decreases the protein levels of p-Akt and CDK2 in MCF7 and MDA-MB-231 cells. After synchronization, the levels of p-Akt and CDK2 were notably reduced at five time points in RHBDD1-knockout cells compared with the wild-type cells (Fig. 5b and c). A widely accepted model for the regulation of cell cycle related proteins is the proteolytic pathway mediated by proteasome [54]. We observed that CDK2 protein level of RHBDD1-knockout cells is restored significantly when treated with MG-132, indicating that CDK2 is degraded by proteasomal pathway after RHBDD1 deletion (Fig. 5d). Consistent with the biochemical results, our immunohistochemical analysis of breast cancer tissue microarrays showed significant positive correlation of RHBDD1, p-Akt and CDK2 protein levels, indicating that these proteins have clinicopathological relevance.

Our previous work demonstrated that proTGFα could be cleaved and released by RHBDD1, and the secretion of TGFα further activates the EGFR-mediated Raf/MEK/ERK signaling pathway [20]. Therefore, we established RHBDD1- TGFα- EGFR axis, though to some extent, this result seems to contradict the result that RHBDD1 triggers non-canonical secretion of TGFα [55]. As Akt-mediated signaling pathways are downstream of EGFR, we speculated that Akt-mediated pathways may be influenced by the decrease of RHBDD1 protein level via the RHBDD1-TGFα-EGFR axis. Accordingly, we proved that RHBDD1 deletion leads to the decrease of p-Akt level. Moreover, previous data reported that reduced p-Akt level down-regulates the expression of CDK2, although the underlying mechanism has not been described [56]. We speculated that the decrease in p-Akt level by RHBDD1 deletion promotes CDK2 degradation via proteasome pathway, leading to G1/S arrest and delayed cell cycle progression. However, there is still much to understand mechanistically about how RHBDD1 regulates p-Akt and CDK2 protein levels and the molecular mechanisms will be evaluated in the future.

## Conclusion

Our data demonstrate the clinical importance of RHBDD1 up-regulation in breast tumor tissue. Besides, we reveal that deletion of RHBDD1 protects breast cancer cells from tumor progression by promoting anti-tumoral properties such as apoptosis, and inhibiting pro-tumoral properties such as proliferation, migration and invasion, as well as cell cycle progression via regulating p-Akt/CDK2 signaling pathway. Though in-depth mechanisms remain to be elucidated, our study provides a preliminary basis to investigate RHBDD1 as a potential therapeutic target for breast cancer treatment.

## Additional files

Additional file 1: **Table S1.** The primers that were used in quantitative real-time PCR reaction. (XLS 9 kb)

Additional file 2: **Table S2.** Differentially expressed genes were listed in MCF7 RHBDD1 knock-out cells versus wild-type cells. (XLS 500 kb)

Additional file 3: **Figure S1.** Kaplan–Meier survival analysis of the correlation between RHBDD1 expression and relapse-free or overall survival in different subtypes of breast cancer patients. a. The correlation between RHBDD1 expression and overall survival in ER positive breast cancer patients (*n* = 109, *p* = 0.039, log-rank test). b. The correlation between RHBDD1 expression and relapse-free survival in ER positive breast cancer patients (*n* = 762, *p* = 0.087, log-rank test). c. The correlation between RHBDD1 expression and relapse-free survival in ER and PR positive breast cancer patients (*n* = 467, *p* = 0.011, log-rank test). d. The correlation between RHBDD1 expression and relapse-free survival in HER2 positive breast cancer patients (*n* = 150, *p* = 0.031, log-rank test). e. The correlation between RHBDD1 expression and relapse-free survival in PR positive breast cancer patients (*n* = 489, *p* = 0.02, log-rank test). f. The correlation between RHBDD1 expression and relapse-free survival in triple negative breast cancer patients (*n* = 161, *p* = 0.03, log-rank test). (TIF 356 kb)

Additional file 4: **Figure S2.** The impact of RHBDD1 knock-down on proliferation rate of HEK 293 T cells. Cell proliferation assay and western blot assay of HEK 293 T cells. The sequences of two siRNA oligos targeting RHBDD1 in the siRNA pool were GUAGAUGGUUUGCCUAUGUTT and GGAUUCUUGUUGGACUAAUTT. Each point in the growth curve represented the mean value of five independent samples. GAPDH was a loading control. Experiments were repeated three times. (TIF 84 kb)

Additional file 5: **Figure S3.** The effect of RHBDD1 deletion on colony formation in breast cancer cells. Representative photos and column graphs are shown from three independent experiment (means±s.d., t test, * *p* < 0.05; ** *p* < 0.01). Experiments were repeated three times. (TIF 182 kb)

Additional file 6: **Table S3.** In MCF7 RHBDD1 knock-out cells, 8 upregulated genes that promoted the apoptotic process and 22 downregulated genes that inhibited the apoptotic process were listed. (XLS 31 kb)

Additional file 7: **Table S4.** 73 genes were differentially expressed between RHBDD1 knock-out and wild-type MCF7 cells. (XLS 16 kb)

Additional file 8: **Table S5.** Patient characteristics in 84 breast cancer specimens. (XLS 8 kb)

## Abbreviations

CDK2: Cyclin-dependent kinase 2
CHX: Cycloheximide
DCIS: Ductal carcinoma in situ
ER: Estrogen receptor
ERAD: Endoplasmic reticulum-associated degradation
FACS: Fluorescence Activated Cell Sorter
GSEA: Gene Set Enrichment Analysis
HER2: Human epidermal growth factor receptor 2
IBC: Invasive breast cancer
IHC: Immunohistochemical analysis
p-Akt: Phospho-Akt
PBS: Phosphate-buffered saline
PI3K: Phosphatidylinositol-4, 5-bisphosphate 3-kinase
pN: Pathological node stage
pT: Pathological tumor stage
pTNM: Pathological tumor-node-metastasis stage
RHBDD1: Rhomboid domain-containing protein 1
RHBDF1: Human rhomboid family 1
TdR: Thymidine
TMA: Tissue microarray
TNBC: Triple-negative breast cancer
TUNEL: TdT-mediated dUTP nick-end labeling
