# Supplementary figures and images for: Rhomboid domain-containing protein 1 promotes breast cancer progression by regulating the p-Akt and CDK2 levels
Source: Cell Commun Signal. 2018 Oct 4;16:65. doi: 10.1186/s12964-018-0267-5 (PMC6172813; doi:10.1186/s12964-018-0267-5)

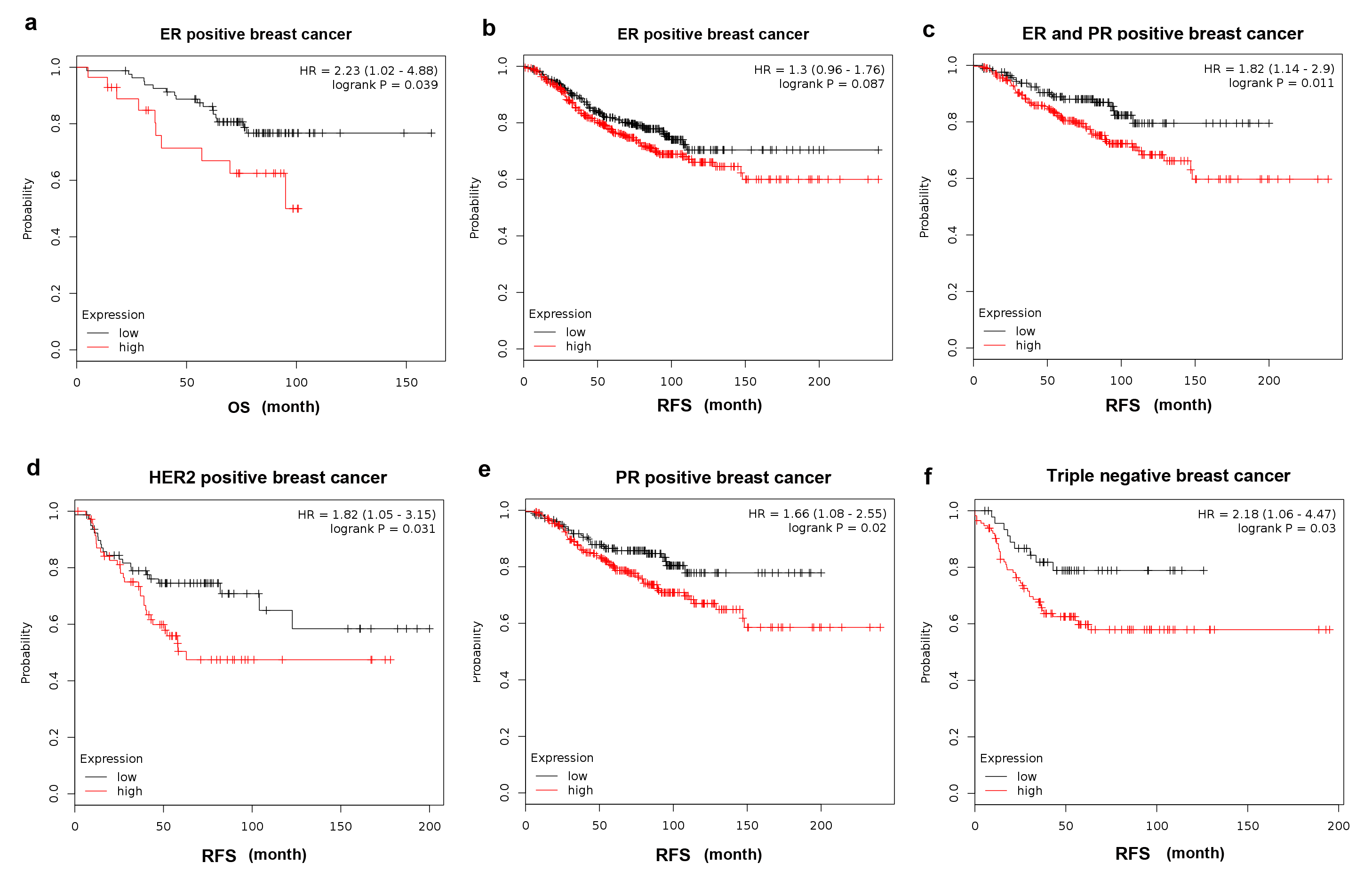

Supplement: Supplementary file 3 — Figure S1. Kaplan–Meier survival analysis of the correlation between RHBDD1 expression and relapse-free or overall survival in different subtypes of breast cancer patients. a. The correlation between RHBDD1 expression and overall survival in ER positive breast cancer patients (n = 109, p = 0.039, log-rank test). b. The correlation between RHBDD1 expression and relapse-free survival in ER positive breast cancer patients (n = 762, p = 0.087, log-rank test). c. The correlation between RHBDD1 expression and relapse-free survival in ER and PR positive breast cancer patients (n = 467, p = 0.011, log-rank test). d. The correlation between RHBDD1 expression and relapse-free survival in HER2 positive breast cancer patients (n = 150, p = 0.031, log-rank test). e. The correlation between RHBDD1 expression and relapse-free survival in PR positive breast cancer patients (n = 489, p = 0.02, log-rank test). f. The correlation between RHBDD1 expression and relapse-free survival in triple negative breast cancer patients (n = 161, p = 0.03, log-rank test). (TIF 356 kb) [file 12964_2018_267_MOESM3_ESM.tif]

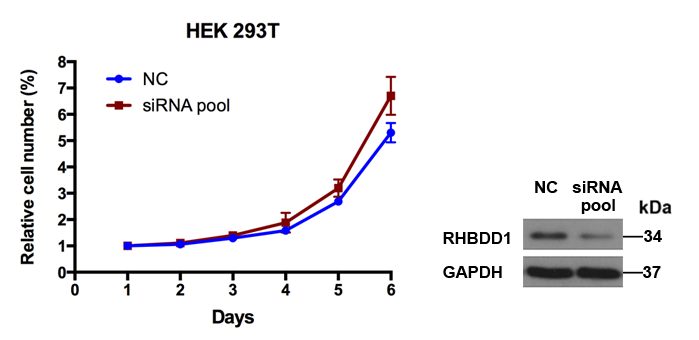

Supplement: Supplementary file 4 — Figure S2. The impact of RHBDD1 knock-down on proliferation rate of HEK 293 T cells. Cell proliferation assay and western blot assay of HEK 293 T cells. The sequences of two siRNA oligos targeting RHBDD1 in the siRNA pool were GUAGAUGGUUUGCCUAUGUTT and GGAUUCUUGUUGGACUAAUTT. Each point in the growth curve represented the mean value of five independent samples. GAPDH was a loading control. Experiments were repeated three times. (TIF 84 kb) [file 12964_2018_267_MOESM4_ESM.tif]

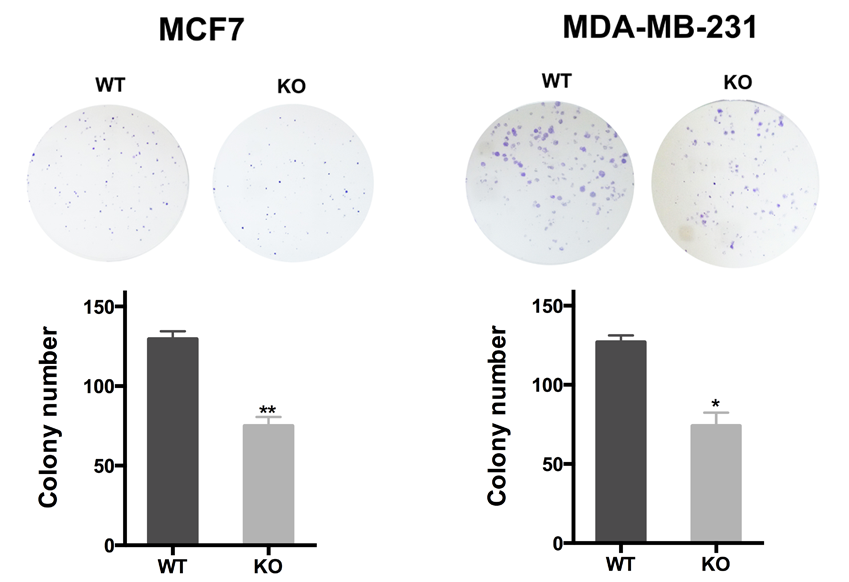

Supplement: Supplementary file 5 — Figure S3. The effect of RHBDD1 deletion on colony formation in breast cancer cells. Representative photos and column graphs are shown from three independent experiment (means±s.d., t test, * p < 0.05; ** p < 0.01). Experiments were repeated three times. (TIF 182 kb) [file 12964_2018_267_MOESM5_ESM.tif]
